# The Development and Characterization of a Novel Prickly Pear–Grape Distilled Spirit

**DOI:** 10.3390/foods15050953

**Published:** 2026-03-08

**Authors:** Artemis P. Louppis, Michalis S. Constantinou, Ioanna S. Kosma, Anastasia V. Badeka, Michael G. Kontominas

**Affiliations:** Laboratory of Food Chemistry, Department of Chemistry, University of Ioannina, 45110 Ioannina, Greece

**Keywords:** prickly pear, fermentation, distilled beverage, antioxidant activity, mineral content, volatiles, sensory attributes

## Abstract

A novel distilled alcoholic beverage was produced by fermenting yellow and red prickly pear (*Opuntia ficus-indica*) fruits with two Cypriot grape varieties (*Mavro* and *Xynisteri*), followed by traditional distillation. Two spirit variants (45% and 59% *v*/*v* alcohol) were prepared and assessed for physicochemical properties, antioxidant capacity, methanol, phenolic and flavonoid content, mineral composition, volatile profile, and sensory characteristics. Both spirits exhibited a pH of 3.83, total titratable acidity of 0.113% (expressed as citric acid), and methanol content between 0.38–1.85 g/hL of 100% *v*/*v* alcohol. Prickly pear addition enhanced the bioactive composition, with the yellow variant showing the highest flavonoid content (5.56 mg/L quercetin) compared to control zivania. Antioxidant activity (FRAP assay) ranged from 1.00 to 1.49 mg FeSO_4_/L. Mineral analysis revealed elevated manganese, cobalt, and nickel in yellow (59% *v*/*v*) spirits, while red variants contained higher aluminum, platinum and magnesium. Volatile profiling showed increased ester and alcohol levels in 59% *v*/*v* beverages, with yellow spirits enriched in fruity esters (e.g., ethyl acetate). Sensory testing confirmed a greater consumer preference for prickly pear beverages, particularly yellow (59% *v*/*v*), which achieved a score of 9.7/10 for overall acceptability. These findings highlight the potential of prickly pear to contribute to the chemical composition and sensory complexity of grape-based distilled spirits.

## 1. Introduction

The global alcoholic beverage market plays a significant role in world economic growth, contributing to employment, government tax revenues, and global GDP. In 2022, the spirits industry generated a world gross added value of $730 billion. The sector also supported 36 million jobs worldwide, spanning production, supply chains, and consumer markets. Additionally, alcoholic beverage manufacturers and related activities contributed $390 billion in tax revenues, making the industry one of the largest tax contributors globally. The market for alcoholic beverages continues to expand, driven by shifting consumer preferences, and increasing demand for high-quality products. In 2022, spirits represented the largest share of alcoholic-beverage sales by value (40%), ahead of beer (38.1%) and wine (17.6%) [1].

Fermentation is considered one of the oldest methods of food preservation. There are various types of fermentation, such as alcoholic, acetic and lactic acid fermentation, which are carried out by microorganisms, including bacteria and yeasts, that are either naturally present in the products or added prior to the fermentation process [2]. One of the applications of fermentation is the production of alcoholic beverages. Alcoholic fermentation is the process of converting sugars into ethanol and carbon dioxide through the action of specific yeasts. This process plays a crucial role with regard to the sensory characteristics of alcoholic beverages, influencing product aroma, taste and texture/body [3]. For the production of distilled spirits, alcoholic fermentation is followed by distillation. Distilled spirits are typically derived from raw materials including grains or fruits, producing brandy, gin, rum, and whiskey, as well as traditional distilled products from different countries, such as tsipouro in Greece, grappa in Italy, and zivania in Cyprus, originating from grapes [4,5,6,7,8,9]. Grape pomace distillation is carried out using fermented grape pomace (skins, seeds, and residual pulp remaining after pressing), whereas wine distillation uses the fermented liquid phase (wine) as the feedstock. Because pomace is a semi-solid matrix with a higher solids content, its distillation typically requires more careful heating/handling than wine to ensure uniform heat transfer and avoid scorching. Consequently, pomace-based distillates are generally more influenced by skin/seed contact during fermentation and processing, while wine distillates primarily reflect the volatile profile of the wine [1,2]. Zivania or zivana is a Cypriot spirit produced through the distillation of grape pomace combined with local dry wines made from the *Xynisteri* and *Mavro* grape varieties. In 2018, zivania was officially registered as a Protected Geographical Indication (PGI) product (PGI-CY-01942, Ref. Ares (2018) 2463635—10 May 2018). Zivania is characterized by an alcohol content ranging from 43% to 52%. It is a clear liquid of strong aromatic intensity, featuring spicy and amylaceous notes, reminiscent of over-ripe grapes, raisins, and spices. Its texture and flavor are defined by a warm, rich, and viscous mouthfeel, attributed to its high ethanol content. Zivania holds significant cultural and economic importance in Cyprus, representing a traditional spirit that not only reflects local viticultural heritage but also supports regional production and identity through its PGI status in addition to exports abroad.

Prickly pear (*Opuntia ficus-indica*) is a nutritionally rich fruit, with a high content of bioactive compounds, including polyphenols, flavonoids, betalains, and vitamin C. It is widely cultivated and naturalized across arid and semi-arid regions, particularly in central America and the Mediterranean basin, and is commonly grown in Cyprus, where the fruits are consumed fresh and also used for the preparation of local food products such as jams/fruit preserves, juices, and traditional beverages [3]. It is also a good source of minerals such as calcium, magnesium, and potassium, as well as dietary fiber. The natural sugars found in prickly pear, including glucose and fructose, make it an excellent substrate for alcoholic fermentation, providing the necessary fermentable sugars for yeast growth [10]. Additionally, its distinct aromatic profile, characterized by fruity and floral notes, may enhance the sensory complexity of fermented alcoholic beverages [11]. The use of prickly pear in fermentation presents an opportunity to create a novel distilled spirit, combining the traditional distillation techniques of Cypriot grape-based spirits with the unique flavor and bioactive properties of the fruit.

Based on the above, the objective of the present study was to produce, via alcoholic fermentation followed by distillation, a novel distilled spirit by combining prickly pear (*Opuntia ficus-indica*) with the Cypriot grape varieties *Mavro* and *Xynisteri*, and to characterize the final products. Conventional physicochemical parameters, volatile profile, mineral content, antioxidant properties (phenolic and flavonoid content), and sensory attributes of the final products were determined. To the best of our knowledge, no previous study has reported a zivania-alternative distilled spirit produced by combining *Opuntia ficus-indica* (prickly pear) with the indigenous Cypriot grape varieties *Mavro* and *Xynisteri*. Therefore, the novelty of this work is the introduction of this local raw-material combination for distillation, along with a comprehensive characterization of the final spirits (physicochemical parameters, volatile profile, minerals, antioxidant indices, and sensory attributes).

## 2. Materials and Methods

### 2.1. Samples

Twenty kilograms of *Opuntia ficus-indica* (10 kg of each the yellow and red cultivar) and grapes (of *Mavro*, a red grape cultivar and of *Xynisteri*, a white grape cultivar) originating from the Limassol prefecture in Cyprus were used. After removing the skin of prickly pears, all fruits were gently washed with tap water and blended for 30 s using a home-type blender (Sirman S.p.A., Curtarolo, Italy).

### 2.2. Chemicals and Reagents

Quercetin (PubChem CID: 5280343) and gallic acid (PubChem CID: 370) (purity > 94%) were obtained from Sigma (St. Louis, MO, USA). Certified single-element stock solutions were supplied by CPAChem (Bogomilovo, Bulgaria): Ca, Mg, P and Cu at 1000 mg/L in 5% HNO_3_, and Li, Sr, V, Mn, Mo, Fe, Co, Ni, Pt, Al and Sb at 100 mg/L in 5% HNO_3_. Hydrogen peroxide (30%, analytical grade) was purchased from Supelco (Belefonte, PA, USA), and ultrapure nitric acid (67–69%, trace analysis grade) from Carlo Erba (Val-de-Reuil, France). Glacial acetic acid (100%, anhydrous), methanol, iron(II) sulfate heptahydrate (FeSO_4_·7H_2_O; PubChem CID: 62662) and anhydrous sodium carbonate (Na_2_CO_3_; PubChem CID: 10340) were sourced from Merck (Darmstadt, Germany). Aluminum chloride hexahydrate (AlCl_3_·6H_2_O, 99%; PubChem CID: 24564), Folin–Ciocalteu phenol reagent (2 M; PubChem CID: 516996) and TPTZ (2,4,6-tris(2-pyridyl)-s-triazine, ≥98%; PubChem CID: 77258) were purchased from Sigma (St. Louis, MO, USA), whereas Trolox (±-6-hydroxy-2,5,7,8-tetramethylchromane-2-carboxylic acid; PubChem CID: 40634) was obtained from Aldrich (St. Louis, MO, USA). Analytical standards for 1-hexanol, 2-nonenol, hexanal, 2-hexenol, 2-nonenal, 2-octanone, and 1,8-cineole were purchased from Supelco (Belefonte, PA, USA); additional fragrance-grade compounds (e.g., 2-hexenol, 3-pentanone and 2-pentylfuran) were acquired from Sigma-Aldrich (St. Louis, MO, USA).

### 2.3. Alcoholic Fermentation

Mixture of grapes (*Mavro* and *Xynisteri*, 10:1) were crushed using a hand-operated grape crusher. The grape must (13.2 °Brix), yellow prickly pear pulp (12.8 °Brix), and red prickly pear pulp (12.9 °Brix) were collected in three separate polyethylene terephthalate (PET) containers of capacity 50 L (Lordos United Plastics Public Ltd., Limassol, Cyprus) for fermentation at T ~ 25 °C and RH ~ 67% in a controlled temperature and humidity room. Three different anaerobic alcoholic fermentations, one for each of the grape must, yellow prickly pear pulp and red prickly pear pulp, were completed in 35 days. Alcoholic fermentation was carried out spontaneously, without inoculation of commercial yeast, relying exclusively on the indigenous microbiota naturally present in the prickly pear fruits and grapes. The end of alcoholic fermentation was determined by monitoring Brix degrees and was considered complete when °Brix values stabilized over two consecutive measurements. All three fermented products were filtered through Filter paper, No185 mm (Macherey-Nagel, Duren, Germany). The fermented grape product was mixed with each of the two prickly pear fermented products in a (1:0.5) ratio. Preliminary tests showed that the optimum sensory results were achieved by the 1:0.5 (*v*/*v*) ratio. Both fermented samples were subjected to traditional batch distillation (85–95 °C) in a copper alembic still, consisting of a pot, swan neck, and condenser, to produce a zivania-alternative distilled spirit. Distillation was carried out in two successive runs: the first to obtain the raw distillate and the second to refine the product by discarding the first and last fractions (heads and tails) and collecting only the middle fraction (heart), which contained the desired ethanol and volatile compounds. The same procedure was performed using only the fermented grape product to produce conventional zivania distilled spirits (control samples). The 1:0.5 (*v*/*v*) ratio was selected to maintain a zivania-type formulation in which the grape component (*Mavro*/*Xynisteri*) remains predominant, while incorporating prickly pear at a lower proportion to contribute aroma and complexity without masking the grape-based character. All final distillates were adjusted to the target alcoholic strength, packaged in 750 mL glass bottles sealed with natural corks, and stored at room temperature until analysis.

### 2.4. Determination of pH, % Alcohol, Titratable Acidity (TA) and Methanol Content

The pH values of the samples were measured using a pH meter (Model FiveGo F2, Mettler-Toledo GmbH, Greifensee, Switzerland) with a precision of ±0.01. All measurements were conducted at 20 ± 1 °C until a constant value was reached. The % alcohol was measured using an alcohol meter (0530FC035/20-QP, Alla, Chemillé-en-Anjou, France). Titratable acidity (TA) was determined according to the official AOAC method of analysis [12] and the results were expressed as % citric acid. Methanol content was determined according to Kokoti et al. [13]. Samples were diluted with ultrapure water and analyzed by gas chromatography with mass spectrometry (Agilent Technologies model 7890A/5975C, Wilmington, DE, USA). Quantification was achieved through external calibration with certified methanol standards.

### 2.5. Determination of Total Phenolic Content (TPC), Total Flavonoid (TFC) and Total Antioxidant Activity (TAA)

Total phenolic content (TPC) was quantified using the Folin–Ciocalteu assay following Singleton et al. [14]. An aliquot of sample (200 μL) was combined with Folin–Ciocalteu reagent (200 μL), 8% (*w*/*v*) Na_2_CO_3_ (1.0 mL), and distilled water (1.6 mL), vortex-mixed for 1 min, and left to react for 30 min. Absorbance was recorded at 765 nm and results were reported as mg gallic acid equivalents (mg GAE/L) of sample. Total flavonoid content (TFC) was determined according to Matić et al. [15]. Briefly, distilled water (800 μL), sample (200 μL) and 5% NaNO_2_ (60 μL) were mixed in a cuvette; after 5 min, 10% AlCl_3_ (60 μL) was added, followed 6 min later by 1 mol/L NaOH (400 μL) and distilled water (480 μL). Absorbance was measured at 510 nm (JASCO V-730, Tokyo, Japan). and TFC was expressed as mg quercetin equivalents (mg QE/L) of sample. Total antioxidant activity (TAA) was assessed by the FRAP assay as described by Ozgen et al. [16] with modifications. FRAP reagent (3.6 mL; TPTZ:FeCl_3_:CH_3_COOH, 1:1:10) was added to 400 μL of sample, vortexed for 1 min, incubated at 37 °C for 10 min, centrifuged (10,000 rpm, 5 min), and filtered (PTFE, 13 mm, 0.2 μm) prior to reading absorbance at 593 nm. Quantification was performed using an FeSO_4_ calibration curve, and results were expressed as mg FeSO_4_/L of sample.

### 2.6. Determination of Mineral Content

A 0.5000 ± 0.0010 g aliquot of each sample was placed in a Teflon microwave-digestion vessel. Hydrogen peroxide (30%, 1 mL) and ultra-pure nitric acid (67–69%, 9 mL) were added, the vessels were sealed, and digestion was performed using an ETHOS EASY microwave system (Milestone, Bergamo, Italy). The program started at 25 °C, increased linearly to 210 °C, and held at 210 °C for 15 min. After cooling, the digests were quantitatively transferred to 20 mL volumetric flasks and brought to volume with deionized water.

Elemental analysis was carried out by ICP-MS (NexION 1000, PerkinElmer, Waltham, MA, USA) fitted with a glass cyclonic spray chamber, a MEINHARD glass Type C nebulizer, a nickel skimmer cone, and an S10 autosampler (PerkinElmer, Waltham, MA, USA). Operating conditions were: nebulizer gas 1.02 L/min, auxiliary gas 1.2 L/min, plasma gas 15 L/min, RF power 1600 W, analog stage voltage −1637 V, cell entrance/exit voltages −6/−32 V, sweep 20, and 3 replicates. Fifteen macro- and trace elements were quantified in the distilled spirits. Based on in-house optimization with certified standards, Ca, P, Mg, and Fe were measured in standard mode, whereas Cu, Li, Sr, V, Mn, Mo, Co, Ni, Pt, Al, and Sb were acquired in KED mode. Quantification relied on the most abundant isotopes; no major spectral interferences were detected, and correction equations were applied where needed (e.g., Fe: −0.028226 + ^52^Cr; Mo: −0.109613 + ^101^Ru).

### 2.7. Determination of Volatile Compounds

The determination of volatile compounds was carried out according to the method described by Kokoti et al. [13]. Briefly, 2.5 mL of sample were mixed with 2.5 mL of deionized water, 1 g of NaCl, and 40 μL of internal standard (4-methyl-2-pentanone). Volatile compounds were then analyzed using solid phase microextraction-gas chromatography-mass spectrometry (SPME–GC–MS, 7890A GC, 5975C MS, Agilent, Santa Clara, CA, USA).

### 2.8. Sensory Evaluation (SE)

Sensory evaluation of the alcoholic beverages (consumer acceptability test) was conducted by a 101-member untrained panel consisting of graduate students and faculty members from the Laboratory of Food Chemistry and Technology, Department of Chemistry, University of Ioannina, Greece ([female 60%/male 40%], adults ≥18 years). Samples were served blind-coded in glass cups covered with a watch glass and were presented to panelists in a randomized order. Prior to evaluation, the samples were allowed to stand for 30 min to allow equilibration of volatile compounds in the glass headspace. The testing was performed in individual booths under controlled conditions of light, temperature, and relative humidity. Panelists were asked to evaluate the color/appearance, odor, and taste of the spirits using a 10-point scale, where 10 represented the most liked sample, equivalent to the primary control sample, and 1 represented the least liked sample (unacceptable). A score of 5 was considered the lower threshold for product acceptability. All samples were placed in glass cups covered with a watch glass. Before evaluation, samples were left to stand for 30 min. to equilibrate headspace volatiles. Scores were recorded for odor, taste, and color/appearance.

### 2.9. Statistical Analysis

A total of six final distillate batch samples were prepared (yellow prickly pear-based distillate, a red prickly pear-based distillate, and control spirits standardized to two alcohol strengths (45% *v*/*v* and 59% *v*/*v*)). The fermentation/distillation experiment was replicated twice on different occasions with triplicate determinations per analytical parameter evaluated per replicate (*n* = 2 × 3 = 6) for each of the six batches of samples prepared. Data were subjected to One-Way ANOVA analysis. Mean differences were evaluated using Tukey’s post hoc test, with statistical significance set at *p* < 0.05.

## 3. Results and Discussion

### 3.1. pH, Ethanol and Methanol Content of Distilled Spirits

The alcohol content of the distillate was adjusted by the addition of distilled water. This process resulted in two groups of zivania-alternative distilled spirits with alcohol content of 45% and 59% *v*/*v*. For clarity, samples are hereafter coded as C59 and C45 (controls), R59 and R45 (red prickly pear spirits), and Y59 and Y45 (yellow prickly pear spirits), where the number denotes the final alcohol strength (% *v*/*v*). The total number of samples evaluated per replicate was six (yellow prickly pear-based distillate, red prickly pear-based distillate and control sample of 45% alcohol content and another three of 59% alcohol content). The alcohol content of the distillates was 45.1%, 45.0%, and 45.0% *v*/*v* for the low alcohol content samples, and 59.0%, 59.1%, and 59.0% *v*/*v* for the high alcohol content samples respectively. The corresponding specific gravity values were 0.921, 0.915, and 0.910 g/mL and 0.940, 0.926, and 0.932 g/mL, respectively. In the zivania-alternative spirit samples, the pH value was 3.83 ± 0.05, and the acidity was 0.113 ± 0.013%, expressed as citric acid. In the control samples, the pH value was 3.85 ± 0.02, and the titratable acidity was 0.111 ± 0.008%, expressed as citric acid with no statistically significant differences being recorded among samples. Statistically significant differences (*p* < 0.05) were observed in methanol concentrations between the low and high alcohol content samples. Methanol levels in the low-alcohol samples were 1.42, 0.68, and 0.38 g/hL of 100% *v*/*v* alcohol for the yellow prickly pear-based spirit, the red prickly pear-based spirit and the control spirit, respectively, while the corresponding values for the high-alcohol samples were 1.85, 0.88, and 0.50 g/hL. The observed variations in methanol concentration for a given alcohol content may be attributed to differences in pectin content of the fruit used, which in turn is influenced by both the fruit variety and its geographical origin. Pectin degradation, facilitated by pectinolytic enzymes that cleave methoxyl groups from pectin molecules in crushed fruits, is the documented pathway for methanol formation. Additionally, factors such as the type of processing and the duration of fruit pulp extraction can further impact methanol production [17,18]. Furthermore, the differences in methanol concentration between low and high alcohol content samples may be attributed to the dilution of the initial distilled product with water to achieve the target alcohol content (*v*/*v*). Despite such differences, all samples complied with EU safety regulations, with the maximum permitted methanol level set at 30 g/hectoliter of 100% *v*/*v* alcohol [19].

Tsegay et al. [20] optimized fermentation conditions for the production of cactus pear wine, resulting in higher titratable acidity (12.39 g/L tartaric acid ≈ 1.057% citric acid). In contrast, the distilled spirit (zivania-alternative) produced in the present work had a significantly lower titratable acidity (0.111–0.113% citric acid). Furthermore, Tsegay et al. [21] analyzed the organic acid composition of cactus pear and *Lantana camara* wines, reporting a higher total titratable acidity (1.06% and 0.83% *w*/*v* citric acid, respectively) compared to the levels determined in the present study. Kokoti et al. [13] studied the effect of distillation technique on the volatile aroma compounds of distilled “tsipouro” spirits. The methanol content in two samples with 42% *v*/*v* alcohol was 3.05 and 4.61 g/hL of 100% *v*/*v* alcohol, which is higher than the levels observed in the present study (0.38–1.85 g/hL of 100% *v*/*v* alcohol for zivania (control) samples). These differences may be explained by the use of different grape varieties, as *Debina* in the referenced study is known for its moderate titratable acidity, compared *Mavro* and *Xynisteri* in the present work.

### 3.2. TPC, TFC and Antioxidant Activity of Distilled Spirits

Total flavonoid and total phenolic content and antioxidant activity (FRAP assay) are shown in Table 1. Calibration curves were constructed in the range of 0.5–10 mg QE/L for flavonoid content, 10–100 mg GAE/L for phenolic content, and 0.1–5 mg FeSO_4_/L for FRAP, with correlation coefficients (R^2^) greater than 0.99 (Supplementary Material, Table S2). Among the three analytical parameters determined, only the total flavonoid content showed significant differences (*p* < 0.05) among the distilled beverages.

The flavonoid content ranged from 1.84 ± 0.02 mg QE/L in the zivania 45% *v*/*v* (control) to 5.56 ± 0.02 mg QE/L in the yellow prickly pear 59% *v*/*v* distilled spirit. The flavonoid levels in the control samples (1.84 ± 0.02 and 4.14 ± 0.02 mg QE/L for 45% and 59% *v*/*v*, respectively) were lower compared to those in the prickly pear distilled spirits. Specifically, the red prickly pear spirits contained 2.09 ± 0.02 mg QE/L at 45% *v*/*v* and 4.49 ± 0.01 mg/L at 59% *v*/*v*, while the yellow prickly pear spirits contained 2.75 ± 0.01 mg QE/L at 45% *v*/*v* and 5.56 ± 0.02 mg QE/L at 59% *v*/*v*. These results indicate that the incorporation of prickly pear, particularly the yellow variety, enhanced flavonoid concentrations. The differences in flavonoid content between the 45% and 59% *v*/*v* distillates may be rationalized as outlined above. Present results are in agreement with those of Tsegay et al. [22], who reported higher levels of total flavonoids in the yellow prickly pear Mexican variety compared to the red variety. Similarly, Butera et al. [23] reported that prickly pears contained twice the amount of total flavonoids as grapes, which may explain why distilled spirits made from prickly pears showed a higher total flavonoid content compared to control samples. In contrast, other physicochemical parameters, including total phenolic content (TPC), did not show statistically significant differences (*p* > 0.05) among the six samples ranging from 29.05 mg GAE/L for R45 to 31.82 mg GAE/L for the C59. With respect to TAA (FRAP assay), values ranged from 1.00 mg FeSO_4_/L for the R45 to 1.49 mg FeSO_4_/L for the Y59.

Tsegay et al. [20] reported antioxidant activity (235.3 mg ascorbic acid/L ~ 11.2 mg FeSO_4_)/L for cactus pear wine. In contrast, the distilled spirits (zivania-alternative) produced in the present work had a lower antioxidant activity, as measured by the FRAP assay (1.00–1.49 mg FeSO_4_/L). Karabagias et al. [11] prepared an alcoholic beverage by fermenting prickly pear juice with honey, containing alcohol levels of 5%, 12%, and 40%, and a total phenolic content (TPC) between 5800 and 9800 mg GAE/L. In the present study the TPC values (29.05–31.82 mg GAE/L) were lower, with this being attributed to the absence of honey and possible phenolic compound losses during distillation. Park et al. [24] reported a significant increase in TPC (419.46–679.18 μg GAE/mL) and total flavonoid content (419.46–679.18 μg QE/mL) in prickly pear following lactic acid fermentation with yuzu peel and guava leaf. In contrast, the present study recorded substantially lower TPC values (29.05–30.27 mg GAE/L), probably due to the absence of these additional ingredients.

### 3.3. Mineral Content

Matrix-matched calibration was performed over 0.1–10 mg/L for all elements, except Mo and Co, which were calibrated over 0.01–1 mg/L; all curves showed coefficients of determination (R^2^) > 0.99 (Supplementary Material, Table S1). Method accuracy was verified by analyzing quality-control samples and a certified reference material (FCCM46-DRA13RM), yielding recoveries of 80–95%. Limits of quantification ranged from 0.001 to 4 mg/L (Supplementary Material, Table S1). Of the 15 elements identified and quantified (Table 2), nine showed statistically significant differences (*p* < 0.05) among the samples. The Y59 alcoholic beverage exhibited significantly higher concentrations (*p* < 0.05) of cobalt (2.73 ± 0.03 μg/kg), manganese (92.25 ± 0.12 μg/kg), nickel (28.01 ± 0.09 μg/kg), and calcium (8.18 ± 0.19 mg/kg) compared to all other samples. Similarly, the Y45 spirit showed elevated levels (*p* < 0.05) of vanadium (3.99 ± 0.14 μg/kg), iron (2.95 ± 0.11 μg/kg), and strontium (65.24 ± 0.12 μg/kg). In contrast, the C59 contained higher concentrations of antimony (1.56 ± 0.03 μg/kg) and platinum (3.50 ± 0.05 μg/kg). These findings suggest that the addition of yellow prickly pear to the distilled spirit enhances the presence of several bioactive minerals (Co, Mn, Ni, Ca, V, Fe, and Sr), potentially contributing to improved functional properties. Furthermore, an increase in alcohol content from 45% to 59% *v*/*v* was associated with higher concentrations of certain minerals, particularly cobalt, manganese, nickel, and calcium, due to the addition of less water in the high alcohol spirit. The presence of these metals may be attributed to multiple sources, including agricultural practices (e.g., fertilizer use leading to soil contamination), processing equipment and storage containers, as well as environmental pollution from industrial activity. According to international food safety regulations (e.g., European Commission Regulation (EC) No. 1881/2006 and its amendments), maximum permissible levels are established for toxic elements such as Pb, Cd, Hg, and As, while elements including Co, Ni, Mn, and Sr are not specifically regulated in distilled spirits but are monitored due to their potential toxicological relevance. Additionally, the use of the dilution water for dilution during distillate preparation could contribute to metal content, as previously reported by Soufleros et al. [17].

### 3.4. Volatile Profile

The volatile compound content of the distilled spirits is presented in Table 3. A total of 28 volatile compounds were identified and semi-quantified in distilled spirits. Twenty-six compounds (eight alcohols, four aldehydes/ketones/acetals, 10 esters, two terpenes, and two hydrocarbons) were determined in the yellow prickly pear beverage 45% *v*/*v*; 27 compounds (eight alcohols, four aldehydes/ketones/acetals, 11 esters, two terpenes, and two hydrocarbons) were determined in the Y59; 25 compounds (eight alcohols, four aldehydes/ketones/acetals, 10 esters, one terpene, and two hydrocarbons) were determined in the R45; 26 compounds (eight alcohols, four aldehydes/ketones/acetals, 11 esters, one terpene, and two hydrocarbons) were determined in the R59; 24 compounds (seven alcohols, four aldehydes/ketones/acetals, 11 esters, one terpene, and one hydrocarbon) were determined in C45 and 26 compounds (seven alcohols, five aldehydes/ketones/acetals, 11 esters, one terpene, and two hydrocarbons) were determined in C59. Analysis of the volatile compounds revealed distinct differences (*p* < 0.05) among yellow, red and control samples at both 45% and 59% alcohol *v*/*v*. Out of the 36 volatiles identified and semi-quantified, 10 showed statistically significant differences (*p* < 0.05) among the samples.

For the 59% alcohol *v*/*v* samples, the total volatile content ranked as follows: yellow prickly pear beverage > red prickly pear beverage > zivania (control), with corresponding values of 459.10, 358.57, and 217.32 mg/L, respectively. Among the higher alcohols, the yellow prickly pear beverage contained the highest concentrations of 2 methyl-1-propanol and 2-methyl-1-butanol, (21.02 and 34.51 mg/L, respectively), followed by the red prickly pear beverage with 16.89 and 25.22 mg/L, respectively, and 8.46 and 17.33 mg/L for the zivania (control). These compounds are known to impart ethereal, alcoholic, fatty, cocoa-like, fusel, leathery, and whiskey-like aromas. The total ethyl alcohol content was ranked as follows: yellow prickly pear beverage > red prickly pear beverage > zivania (control), with corresponding values of 178.31, 130.55, 82.39, mg/L, respectively.

Among the esters, the yellow prickly pear beverage also had the highest concentrations including ethyl acetate (121.98 mg/L), 1-butanol, 3-methyl acetate (7.85 mg/L), ethyl octanoate (41.15 mg/L), ethyl decanoate (56.22 mg/L), and isoamyl octanoate (1.50 mg/L), all contributing fruity and sweet notes. The total ester content was ranked as follows: yellow prickly pear beverage > red prickly pear beverage > zivania (control), with corresponding values of 228.39, 189.67, 124.17, mg/L, respectively. The elevated ester content resulted in a more aromatic and pleasant flavor profile in the beverages.

Additionally, limonene, a compound known for its citrus-like, fruity, and fresh aroma, was detected in all samples, with the highest concentration observed in the yellow prickly pear beverage (12.07 mg/L) followed by the red prickly pear beverage (10.02 mg/L) and zivania (control) with 0.74 mg/L.

For the 45% alcohol *v*/*v* samples, the total volatile content ranked as follows: yellow prickly pear beverage > red prickly pear beverage > zivania (control), with values of 315.03, 241.25, and 158.61 mg/L, respectively. Among the higher alcohols, the red prickly pear beverage contained the highest concentration of 2 methyl-1-propanol, (6.22 mg/L), followed by the zivania (control) with 5.88 mg/L and 5.78 mg/L for the yellow prickly pear beverage. In contrast, the yellow prickly pear beverage contained the highest concentration of the 2-methyl-1-butanol (17.95 mg/L) followed by the red prickly pear beverage with 11.95 mg/L and 9.21 mg/L for the zivania (control). The total alcohol content (1-Propanol, 1-Propanol, 2-methyl- (isobutanol), 1-Butanol, Isoamyl alcohol, 1-Butanol, 3-methyl- (isoamyl alcohol synonym), 1-Butanol, 2-methyl-, 1-Hexanol, Phenylethyl alcohol) was ranked as follows: yellow prickly pear > red prickly pear beverage > zivania (control), with corresponding values of 104.28 and 73.48, 55.53 mg/L, respectively.

Among the esters, the yellow prickly pear beverage had the highest concentrations of key aromatic compounds, including ethyl acetate (108.03 mg/L), ethyl octanoate (30.99 mg/L) and 3-methylbutyl octanoate (1.05 mg/L). However, the zivania (control) had the higher level of ethyl butanoate (0.28 mg/L). The total ester content (Ethyl acetate, Butanoic acid, ethyl ester, Propanoic acid, 2-hydroxy-, ethyl ester (ethyl lactate, Lactic acid), ethyl ester (same as above—ethyl lactate), 1-butanol, 3-methyl-acetate, Hexanoic acid, ethyl ester, Heptanoic acid, ethyl ester, Butanoic acid, ethyl ester, Octanoic acid, ethyl ester, Isopentyl hexanoate, Nonanoic acid, ethyl ester, Decanoic acid, methyl ester, Decanoic acid, ethyl ester, Octanoic acid, 3-methylbutyl ester (isoamyl octanoate)) was ranked as follows: yellow prickly pear beverage > red prickly pear beverage > zivania (control), with corresponding values of 185.53, 147.86, and 97.18 mg/L, respectively.

Additionally, limonene was detected in all samples, with the highest concentration observed in the yellow prickly pear beverage (9.46 mg/L) followed by the red prickly pear beverage (7.45 mg/L) and zivania (control) with 0.35 mg/L.

The total volatile content values of the distillates comply with the minimum requirements for volatile substances in grape marc spirits, as specified by Regulation (EU) 2019/787 [19]. In summary, the 59% *v*/*v* prickly pear distilled spirits, particularly those made from yellow and red varieties, demonstrated a richer and more complex volatile profile compared to the 45% *v*/*v* spirits as expected due to the addition of less water in the final spirit. The levels of volatile compounds are influenced by the grape and prickly pear variety, fermentation conditions and distillation [17]. The higher concentrations of esters, higher alcohols, and aldehydes in the 59% samples maybe explained according to previous logic.

Kokoti et al. [13] studied the effect of distillation technique on the volatile aroma compounds of distilled “tsipouro” spirits. A total of 44 volatile compounds were identified and semi-quantified, a number comparable to the 27 compounds identified in the zivania (control) samples (59% alcohol) of the present study. Esters were the most abundant group of volatiles in tsipouro, consistent with our findings. Finally, dl-limonene was the dominant terpene identified in all tsipouro samples, aligning with the results observed in our distilled spirits. The volatile profile of prickly pear-based distillates cannot be directly compared against the literature, as directly comparable datasets for distilled spirits produced specifically from prickly pear are currently very limited. A general comparison between the volatile profile of the control zivania and the prickly pear-containing distillates can provide a reasonable indication of which aroma-active compounds are primarily associated with prickly pear incorporation in zivania-type distillates. Such a comparison between volatile compounds of the zivania-type distillates and those of *Opuntia ficus-indica* fruit in Louppis et al. [10] was carried out. Specifically, compounds that were consistently enriched in the prickly pear formulations relative to the control included terpenes (dl-limonene) along with esters in line with the characteristic fruity/citrus and floral notes previously described for prickly pear matrices [10]. In this context, the higher total ester content observed in the prickly pear beverages (particularly the yellow formulation) is also consistent with the higher odor and taste acceptability scores, as esters are key contributors to pleasant fruity aroma perception in distilled beverages. Thus, prickly pear acts as a natural source to enhance the sensory profile of the distillate, offering a feasible route to develop a value-added zivania-alternative that retains the traditional concept of grape-based distillation yet provides enhanced sensory appeal and product differentiation.

### 3.5. Sensory Evaluation

Sensory evaluation included the assessment of odor, taste, color, and overall acceptability of the yellow and red prickly pear distilled spirits, in comparison to the control samples (C45 and C59) These results are presented in Table 4. Spirits that exhibited fruity and floral aromas, a pleasant taste, and a distinctive color received the highest scores. Odor, taste, and overall acceptability showed statistically significant differences (*p* < 0.05) among the samples.

For the 59% alcohol *v*/*v* samples, the yellow prickly pear beverage received the highest scores, with 9.6 for odor, 9.6 for taste, and an overall score of 9.7, followed by the red prickly pear beverage with 9.3 for odor, 9.3 for taste, and an overall score of 9.5 and zivania (control) with 8.4 for odor, 8.4 for taste, and an overall score of 8.9. The higher scores of the prickly pear spirits are attributed to the inclusion of fruit-derived compounds.

For the 45% alcohol *v*/*v* samples, the yellow prickly pear beverage also received the highest scores, with 9.3 for odor, 9.3 for taste, and an overall score of 9.5 followed by the red prickly pear beverage with 9.1 for odor, 9.1 for taste, and an overall score of 9.3 and zivania (control) with 8.2 for odor, 8.1 for taste, and an overall score of 8.7.

Overall, the addition of prickly pear significantly enhanced the sensory characteristics of the distilled spirits, with the yellow variety, especially at 59% *v*/*v*, contributing to a more aromatic, flavorful, and well-balanced profile. The data suggest that a higher alcohol content may enhance the expression of key aroma and flavor compounds, resulting in greater consumer acceptance. Finally, the sensory evaluation results are consistent with the findings from the volatile compound analysis.

## 4. Conclusions

In the present study, red and yellow prickly pear (*Opuntia ficus-indica*) in combination with the Cypriot grape varieties *Mavro* and *Xynisteri* were used to develop a novel zivania-alternative distilled beverage. Using the grape-based control as a typical benchmark, prickly pear incorporation provided clear compositional and sensory differentiation, offering a practical route to diversify grape marc spirits with a distinct aromatic profile and improved consumer acceptability. The highest acceptability was observed for the yellow prickly pear spirit at 59% *v*/*v*, consistent with its more intense aroma perception and its volatile-profile pattern. Overall, this approach highlights the potential of integrating locally available raw materials to create value-added, differentiated distilled products aligned with traditional processing. Future work should include larger-scale validation and targeted profiling of intermediate matrices to better elucidate the contribution of each processing step to the final volatile fingerprint.

## Figures and Tables

**Table 1:** Antioxidant content of fruit-based alcoholic beverages (*n* = 6, average values ± standard deviation).

	C59	C45	R59	R45	Y59	Y45
Total flavonoids (mg QE/L)	4.14 ± 0.02 ^d^	1.84 ± 0.02 ^a^	4.49 ± 0.01 ^e^	2.09 ± 0.02 ^b^	5.56 ± 0.02 ^f^	2.75 ± 0.01 ^c^
Total phenolics (mg GAE/L)	31.82 ± 0.03	30.27 ± 0.04	30.73 ± 0.03	29.05 ± 0.02	31.08 ± 0.03	29.32 ± 0.03
FRAP (mg FeSO_4_/L)	1.47 ± 0.01	1.22 ± 0.01	1.27 ± 0.02	1.00 ± 0.00	1.49 ± 0.01	1.24 ± 0.02

^a–f^ Different superscripts in the same row indicate statistically significant differences (*p* < 0.05). C: control (zivania); R: red prickly pear; Y: yellow prickly pear; numbers denote alcohol strength (% *v*/*v*). Absence of superscripts indicates no statistically significant differences.

**Table 2:** Mineral content of fruit-based alcoholic beverages (*n* = 6, average values ± standard deviation).

Εlement	C59	C45	R59	R45	Y59	Y45
Li (μg/kg)	323.83 ± 0.09	313.02 ± 0.09	342.15 ± 0.13	305.03 ± 0.18	397.88 ± 0.13	314.88 ± 0.08
Mo (μg/kg)	119.01 ± 0.09	100.33 ± 0.27	132.01 ± 0.15	114.02 ± 0.09	135.22 ± 0.08	119.02 ± 0.07
Sb (μg/kg)	1.56 ± 0.03 ^e^	0.59 ± 0.04 ^c^	0.69 ± 0.05 ^b^	0.08 ± 0.01 ^d^	0.61 ± 0.04 ^b^	0.40 ± 0.01 ^a^
Pt (μg/kg)	3.50 ± 0.05 ^f^	1.94 ± 0.03 ^e^	1.56 ± 0.04 ^c^	1.02 ± 0.18 ^d^	0.45 ± 0.02 ^b^	n.d ^a^
V (μg/kg)	1.98 ± 0.03 ^a^	1.42 ± 0.04 ^f^	0.53 ± 0.04 ^b^	n.d ^c^	5.04 ± 0.17 ^e^	3.99 ± 0.14 ^d^
Co (μg/kg)	2.04 ± 0.22 ^a^	1.16 ± 0.08 ^c^	2.22 ± 0.11 ^a^	1.94 ± 0.05 ^b^	2.73 ± 0.03 ^d^	1.11 ± 0.03 ^c^
Fe (mg/kg)	2.84 ± 0.05 ^a^	2.54 ± 0.17 ^a^	3.45 ± 0.13 ^b^	3.02 ± 0.54 ^b^	3.88 ± 0.03 ^c^	2.95 ± 0.11 ^b^
Sr (μg/kg)	60.02 ± 0.21 ^a^	55.45 ± 0.15 ^b^	50.88 ± 0.12 ^c^	15.88 ± 0.13 ^d^	72.14 ± 0.10 ^e^	65.24 ± 0.12 ^f^
Al (mg/kg)	3.39 ± 0.19	3.05 ± 0.11	3.58 ± 0.19	3.04 ± 0.08	3.03 ± 0.03	2.19 ± 0.05
Mn (μg/kg)	30.99 ± 0.02 ^a^	28.22 ± 0.05 ^b^	25.17 ± 0.04 ^c^	7.21 ± 0.05 ^d^	92.25 ± 0.12 ^f^	35.04 ± 0.13 ^e^
Ni (μg/kg)	14.55 ± 0.14 ^e^	10.03 ± 0.4 ^d^	10.15 ± 0.05 ^d^	8.94 ± 0.07 ^c^	28.01 ± 0.19 ^b^	4.62 ± 0.04 ^a^
Cu (mg/kg)	3.66 ± 0.08	3.14 ± 0.09	3.48 ± 0.11	3.12 ± 0.07	11.11 ± 0.03	6.11 ± 0.09
P (mg/kg)	6.65 ± 0.09	5.81 ± 0.02	5.89 ± 0.13	5.41 ± 0.18	5.82 ± 0.09	5.09 ± 0.07
Ca (mg/kg)	10.02 ± 0.03 ^b^	7.15 ± 0.07 ^e^	4.13 ± 0.05 ^c^	2.05 ± 0.07 ^a^	8.18 ± 0.19 ^f^	4.32 ± 0.06 ^d^
Mg (mg/kg)	14.33 ± 0.10	13.22 ± 0.09	14.52 ± 0.11	13.15 ± 0.09	13.62 ± 0.09	10.99 ± 0.08

n.d: not detected. ^a–f^ Different superscripts in the same row indicate statistically significant differences (*p* < 0.05). C: control (zivania); R: red prickly pear; Y: yellow prickly pear; numbers denote alcohol strength (% *v*/*v*). Absence of superscripts indicates no statistically significant differences.

**Table 3:** Volatile content, mg/L (*n* = 6, average values ± standard deviation) of fruit-based alcoholic beverages.

R.T. (min)	RI_exp_ *	RI_lit_ **	Compound	C59	C45	R59	R45	Y59	Y45
**Alcohols**									
6.166	552	554	1-Propanol	2.22 ± 0.36	0.99 ± 0.25	2.62 ± 0.13	0.89 ± 0.12	3.15 ± 0.22	1.11 ± 0.09
8.253	627	625	1-Propanol, 2-methyl-	8.46 ± 2.29 ^b^	5.88 ± 0.30 ^a^	16.89 ± 0.82 ^c^	6.22 ± 0.07 ^a^	21.02 ± 0.51 ^d^	5.78 ± 0.41 ^a^
9.394	657	650	1-Butanol	1.16 ± 0.29	0.16 ± 0.02	1.52 ± 0.29	0.10 ± 0.03	2.05 ± 0.05	0.25 ± 0.11
11.771	735	743	Isoamylacohol	20.45 ± 0.14	18.02 ± 0.33	35.32 ± 0.05	18.55 ± 0.09	56.71 ± 0.18	31.33 ± 0.84
11.815	736	743	1-Butanol, 3-methyl-	32.45 ± 1.22	21.11 ± 1.08	46.32 ± 3.14	35.05 ± 5.24	57.08 ± 1.89	46.28 ± 6.005
11.892	739	748	1-Butanol, 2-methyl-	17.33 ± 0.51 ^c^	9.21 ± 0.15 ^b^	25.22 ± 1.02 ^d^	11.95 ± 0.20 ^a^	34.51 ± 0.51 ^e^	17.95 ± 0.19 ^ab^
15.645	868	862	1-Hexanol	n.d	n.d	2.14 ± 0.09	0.38 ± 0.08	3.11 ± 0.11	1.02 ± 0.05
21.962	1133	1121	Phenylethyl Alcohol	0.32 ± 0.04	0.16 ± 0.09	0.52 ± 0.05	0.34 ± 0.03	0.68 ± 0.05	0.56 ± 0.04
**Esters**									
7.711	612	614	Ethyl Acetate	81.53 ± 3.50 ^c^	70.13 ± 1.31 ^b^	109.25 ± 8.32 ^d^	95.82 ± 2.5 ^a^	121.98 ± 0.81 ^ad^	108.03 ± 0.90 ^d^
13.698	799	799	Butanoic acid, ethyl ester	0.51 ± 0.09 ^a^	0.28 ± 0.05 ^b^	0.24 ± 0.02 ^b^	n.d^c^	0.63 ± 0.10 ^d^	n.d^c^
14.067	812	798	Propanoic acid, 2-hydroxy-, ethyl ester; Lactic acid, ethyl ester	0.42 ± 0.05 ^b^	0.09 ± 0.02 ^a^	0.58 ± 0.11 ^b^	0.15 ± 0.03 ^a^	0.78 ± 0.07 ^c^	0.38 ± 0.02 ^d^
15.834	877	877	1-Butanol, 3-methyl-, acetate	1.98 ± 0.08 ^c^	0.86 ± 0.05 ^d^	2.68 ± 0.04 ^c^	0.58 ± 0.03 ^a^	7.85 ± 0.69 ^d^	1.15 ± 0.04 ^ab^
18.868	995	996	Hexanoic acid, ethyl ester	2.39 ± 0.05	1.19 ± 0.06	2.55 ± 0.04	0.15 ± 0.01	3.82 ± 0.07	0.23 ± 0.03
21.083	1094	1097	Heptanoic acid, ethyl ester	0.13 ± 0.02	0.07 ± 0.04	0.11 ± 0.03	0.06 ± 0.01	0.22 ± 0.01	0.11 ± 0.02
23.125	1193	1193	Octanoic acid, ethyl ester	10.11 ± 0.55 ^e^	7.93 ± 0.74 ^f^	28.15 ± 2.30 ^a^	17.55 ± 0.26 ^b^	41.15 ± 2.47 ^c^	30.99 ± 0.03 ^d^
25.034	1293	1294	Nonanoic acid, ethyl ester	0.41 ± 0.08	0.27 ± 0.07	1.68 ± 0.34	0.33 ± 0.04	1.99 ± 0.22	0.55 ± 0.02
25.566	1323	1328	Decanoic acid, methyl ester	0.10 ± 0.02	0.07 ± 0.03	0.18 ± 0.03	0.09 ± 0.01	0.32 ± 0.01	0.12 ± 0.02
26.695	1392	1398	Decanoic acid, ethyl ester	27.93 ± 5.59 ^c^	16.68 ± 6.52 ^b^	45.62 ± 0.80 ^a^	33.02 ± 5.11 ^c^	56.22 ± 2.20 ^d^	44.18 ± 5.48 ^a^
27.473	1448	1450	Octanoic acid, 3-methylbutyl ester	0.77 ± 0.11 ^a^	0.54 ± 0.14 ^b^	1.42 ± 0.09 ^d^	0.75 ± 0.06 ^a^	1.50 ± 0.03 ^e^	1.05 ± 0.05 ^c^
**Aldehydes**									
21.396	1108	1099	Nonanal	0.31 ± 0.10	0.11 ± 0.02	0.19 ± 0.02	0.11 ± 0.04	0.26 ± 0.01	0.15 ± 0.00
**Ketones**									
18.316	973	961	4-Heptanone, 2,6-dimethyl-	0.33 ± 0.08	0.18 ± 0.03	0.39 ± 0.05	0.25 ± 0.02	0.50 ± 0.04	0.31 ± 0.05
**Terpenes**									
18.784	992	992	beta-Myrcene	n.d	n.d	n.d	n.d	0.25 ± 0.04	0.11 ± 0.02
19.971	1044	1035	dl-Limonene	0.74 ± 0.08 ^b^	0.35 ± 0.02 ^a^	10.02 ± 0.62 ^c^	7.45 ± 0.18 ^b^	12.07 ± 0.54 ^c^	9.46 ± 0.15 ^c^
**Furans**									
15.421	859	856	2-(2-propenyl)-furan	0.14 ± 0.03	n.d	n.d	n.d	n.d	n.d
**Aromatic hydrocarbons**									
15.71	869	862	Benzene, ethyl-	0.53 ± 0.05	n.d	0.38 ± 0.04	0.21 ± 0.03	0.44 ± 0.01	0.35 ± 0.06
18.501	968	976	Benzene, 1,3,5-trimethyl-; Mesitylene	1.90 ± 0.14	1.02 ± 0.11	1.92 ± 0.45	0.91 ± 0.18	2.11 ± 0.15	1.22 ± 0.17
**Acetals/Ethers**									
11.449	726	725	Ethane, 1,1-diethoxy-	4.42 ± 0.22	3.15 ± 0.19	22.31 ± 0.51	10.25 ± 0.11	28.12 ± 0.19	12.08 ± 0.35
15.291	854	865	Propane, 1,1-diethoxy-2-methyl-Isobutyraldehyde, diethyl acetal	0.28 ± 0.07	0.16 ± 0.02	0.35 ± 0.03	0.09 ± 0.04	0.58 ± 0.09	0.28 ± 0.06
**Total volatiles**				**217.32**	**158.61**	**358.57**	**241.25**	**459.10**	**315.03**

n.d: not detected. * Experimental retention index, ** literature retention index (NIST MS search). ^a–f^ Different superscripts in the same row indicate statistically significant differences (*p* < 0.05). C: control (zivania); R: red prickly pear; Y: yellow prickly pear; numbers denote alcohol strength (% *v*/*v*). Absence of superscripts indicates no statistically significant differences.

**Table 4:** Sensory evaluation of alcoholic beverages (*n* = 6, average values ± standard deviation).

	C59	C45	R59	R45	Y59	Y45
Odor score	8.4 ± 0.1 ^a^	8.2 ± 0.2 ^a^	9.3 ± 0.2 ^b^	9.1 ± 0.2 ^b^	9.6 ± 0.2 ^c^	9.3 ± 0.1 ^b^
Taste score	8.4 ± 0.2 ^b^	8.1 ± 0.1 ^a^	9.3 ± 0.1 ^b^	9.1 ± 0.1 ^b^	9.6 ± 0.1 ^c^	9.3 ± 0.2 ^b^
Color score	9.9 ± 0.1	9.8 ± 0.2	9.8 ± 0.2	9.7 ± 0.3	9.8 ± 0.1	9.8 ± 0.1
Overall score	8.9 ± 0.1 ^a^	8.7 ± 0.2 ^a^	9.5 ± 0.2 ^b^	9.3 ± 0.2 ^b^	9.7 ± 0.1 ^c^	9.5 ± 0.1 ^b^

^a–c^ Different superscripts in the same row indicate statistically significant differences (*p* < 0.05). C: control (zivania); R: red prickly pear; Y: yellow prickly pear; numbers denote alcohol strength (% *v*/*v*). Absence of superscripts indicates no statistically significant differences.

## Data Availability

The original contributions presented in this study are included in the article/Supplementary Materials. Further inquiries can be directed to the corresponding author.

## Supplementary Materials

The following supporting information can be downloaded at: https://www.mdpi.com/article/10.3390/foods15050953/s1, Table S1: Calibration curves, linear regressions, limit of detections (LODs), recoveries for quality control (QC) samples and CRM sample for minerals. Table S2: Calibration curves and linear regressions for total flavonoid content, total phenolic content and antioxidant activity (FRAP assay).
