# Supplementary material for: The Development and Characterization of a Novel Prickly Pear–Grape Distilled Spirit
Source: Foods. 2026 Mar 8;15(5):953. doi: 10.3390/foods15050953 (PMC12984939; doi:10.3390/foods15050953)
Supplement: Supplementary file 1 [file foods-15-00953-s001.zip › foods-4133474-supplementary.pdf]

Supplementary material

Table S1. Calibration curves, linear regressions, limit of detections (LODs), recoveries for quality control (QC) samples and CRM sample for minerals.

**Mineral**	**Calibration curve**	**Linear Regression (R^2)**	**LOD (mg /L)**	**Recovery of QC (%)**	**Recovery of CRM (%)**
K	Y=22868x	0.999948	4	98	-
Ca	Y=26257x	0.999957	4	92	-
P	Y=3257273x	0.999963	4	99	-
Mg	Y=17253x	0.999949	4	95	-
B	Y=7892x	0.998653	0.1	89	-
Si	Y=101x	0.991357	0.1	90	-
Zn	Y=476x	0.997623	0.1	89	-
Mn	Y=758x	0.999889	0.1	88	-
Na	Y=71x	0.990118	0.1	90	-
Fe	Y=3080x	0.999685	0.1	92	95
Sr	Y=3163x	0.999927	0.1	95	-
Al	Y=78071x	0.998833	0.1	99	-
Cu	Y=5390x	0.999532	0.1	90	92
Ni	Y=1958x	0.999983	0.1	97	-
Ba	Y=117484x	0.999333	0.1	98	-
Sn	Y=532x	0.999952	0.001	89	88
Ti	Y=171x	0.997782	0.001	88	-
Mo	Y=60408x	0.998268	0.001	85	-
Co	Y=1542x	0.999847	0.001	86	-

Table S2. Calibration curves and linear regressions for total flavonoid content, total phenolic content and antioxidant activity (FRAP assay).

	**Calibration curve**	**Linear Regression (R^2)**
FRAP (mg $FeSO_4$ /L)	Y=0.0135x-0.0072	0.9991
Total flavonoid (mg QE/L)	Y=0.0311x-0.0165	0.9993
Total phenolics (mg GAE/L)	Y=0.0078x-0.2215	0.9999
